# Polarization-Dependent Metasurface Enables Near-Infrared Dual-Modal Single-Pixel Sensing

**DOI:** 10.3390/nano13091542

**Published:** 2023-05-04

**Authors:** Rong Yan, Wenli Wang, Yao Hu, Qun Hao, Liheng Bian

**Affiliations:** 1 MIIT Key Laboratory of Complex-Field Intelligent Sensing, Beijing Institute of Technology, Beijing 100081, China; 2 Advanced Research Institute of Multidisciplinary Science & School of Information and Electronics, Beijing Institute of Technology, Beijing 100081, China; 3 Beijing Key Laboratory for Precision Optoelectronic Measurement Instrument and Technology, School of Optics and Photonics, Beijing Institute of Technology, Beijing 100081, China

**Keywords:** infrared single-pixel, metasurface, dual modes, optical computing, biomedical image segmentation

## Abstract

Infrared single-pixel sensing with the two most representative modes, bright-field imaging and edge-enhanced imaging, has great application potential in biomedical diagnosis and defect inspection. Building a multifunctional and miniature optical computing device for infrared single-pixel sensing is extremely intriguing. Here, we propose and validate a dual-modal device based on a well-designed metasurface, which enables near-infrared bright-field and edge-enhanced single-pixel imaging. By changing the polarization of the incident beam, these two different modes can be switched. Simulations validate that our device can achieve high-fidelity dual-modal single-pixel sensing at 0.9 μm with certain noise robustness. We also investigate the generalization of our metasurface-based device and validate that different illumination patterns are applied to our device. Moreover, these output images by our device can be efficiently utilized for biomedical image segmentation. We envision this novel device may open a vista in dual-modal infrared single-pixel sensing.

## 1. Introduction

Benefiting from the superior detection efficiency and low cost of the single-pixel method [1], infrared single-pixel sensing is emerging as an enabling technology of great technical and scientific interest [2,3,4]. It avoids the use of infrared array sensors with large dark current, low resolution, and high manufacturing cost, but uses a spatial light modulator (SLM) to compress and couple spatial information. It has great potential in various applications, such as defect inspection, biomedical diagnosis [5,6], and remote sensing [7,8]. In these envisioned scenarios, fast and reliable multifunctional sensing plays a vital role, especially when different tasks are needed.

Conventional strategies for different sensing are mainly digital domain computations using integrated circuits at the expense of high-power consumption, low speed, and limited capacity [9]. For better application, optical analog computing is proposed to tailor the field of the incident beam by placing appropriate optical elements in the optical system. It also modulates image information without limiting capacity and allows for parallel operation [10]. However, conventional optical analog computing inevitably increases the complexity and bulk of optical systems, especially when different sensing tasks are needed to be performed [11]. Therefore, developing a multifunctional and miniature optical analog computing device for infrared single-pixel sensing is extremely intriguing.

Metasurfaces, as specially designed two-dimensional optical elements, have become research hotspots. The subwavelength structure of the metasurface can interact with the incident electromagnetic field. These special nanostructures can also exert flexible and large-scale modulation on the amplitude, phase, polarization, and other characteristics of the optical field within the subwavelength thickness range [12,13]. This sudden change in optical parameters breaks the dependence of traditional optical elements on the propagation path. They have great potential in different optical wavefront modulations without changing the components of the optical systems [14,15,16,17,18,19,20]. Meta-designed sensors have shown promise with extremely high sensitivity [21,22]. Moreover, a single metasurface can replace several traditional optical elements, further minimizing the optical systems. These potentials have pushed metasurfaces into optical computing research areas [10,23,24] for conducting some practical tasks, such as optical logic operations [25], optical differentiation operations [26], and optical neural networks [27]. Among these tasks, optical differentiation operations based on computing metasurfaces are mainly used for image edge enhancement. These edge-enhanced images containing textures and morphologies can be used for further sensing tasks. Remarkably, they have been successfully validated for different imaging, including switchable edge-enhanced and bright-field imaging [11,28], tunable edge-enhanced imaging by adding an electric power driver [29,30], etc. In another context, the research on applying the metasurface in the single-pixel imaging domain has attracted extensive attention. Various metasurfaces have been studied for their fascinating roles in conventional single-pixel systems [5,31,32,33]. So far, a metasurface that can host phase-only and helicity-dependent holograms has been proposed to work as switchable and secret ghost imaging targets. This work builds the first bridge between the metasurface hologram and single-pixel imaging [5]. A metasurface can also be a prototype SLM for high-frame-rate single-pixel imaging or simplifying the single-pixel imaging system [31,32]. These works provide new applications for single-pixel imaging. More interestingly, a novel optical encryption scheme has also been proposed depending on the combination of metasurface hologram and single-pixel imaging technology [33]. The metasurface for dual-modal single-pixel sensing is valuable but has not received sufficient attention. Regardless, the development of these metasurface-based devices brings great hope to our research.

Here, we report a dual-modal metasurface-based device for near-infrared single-pixel sensing, which enables infrared and edge-enhanced imaging as requested. The dual-modal device is mainly achieved by a designed metasurface, which can function as different spatial filters just by rotating the polarizer in this device. Specifically, when the polarization of the incident beam is *y*-linear polarization (YLP), the output mode is high-fidelity bright-field imaging. Similarly, when the polarization of the incident beam is *x*-linear polarization (XLP), the output mode is high-fidelity edge-enhanced imaging. Additionally, this device is suitable for various illumination patterns, and the same illumination pattern can be used in dual modes. Results show that this device can achieve high-fidelity dual-modal single-pixel sensing at 0.9 μm with certain robustness. Moreover, the output images of our device can be efficiently utilized for further computer vision tasks, such as biomedical image segmentation.

## 2. Principle

### 2.1. Principle of the Device

**Figure 1:**
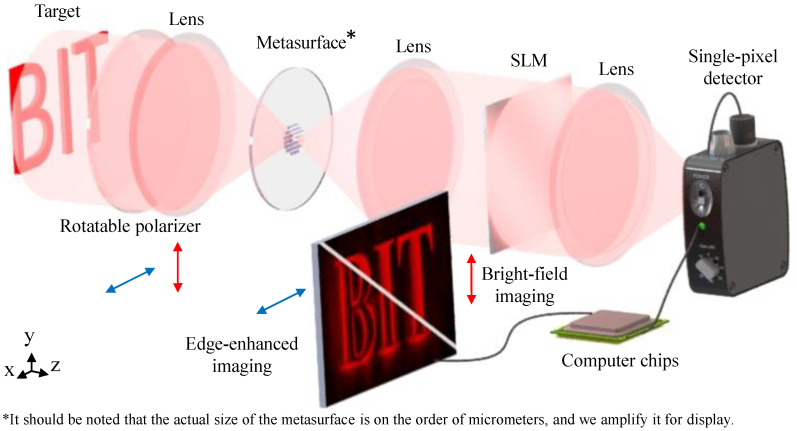
The framework of the near-infrared dual-modal single-pixel sensing metasurface-based device.

Figure 1 is the framework of the proposed device for near-infrared dual-modal single-pixel sensing. This device consists of a 4f imaging system embedded with a polarization-dependent metasurface and classical single-pixel imaging optical elements, which can efficiently obtain bright-field or edge-enhanced images. We can switch these two functions only by rotating the polarizer. Specifically, when the polarization of the incident beam is YLP, and the SLM projects Fourier basis patterns on sequence, the output field is a reconstructed high-fidelity bright-field image. Similarly, when the polarization of the incident beam is XLP, and the SLM projects the same illumination patterns, the output field is a reconstructed high-fidelity edge-enhanced image. In addition, this device is suitable for various illumination patterns, and the images obtained by our device can be used for further computer vision tasks, which we will show later.

To explain the feasibility of our proposed device, we analyze the whole processing and relevant principle in detail. In this system, the target scene is modulated to an XLP or YLP incident beam $E_{in}\left(x,y\right)$ by the rotatable polarizer. *x* and *y* are, respectively, *x*- and *y*-direction coordinates in the input or output image plane. Then spatial filtering is achieved by optical computing. It is contributed by the 4f imaging system embedded with a polarization-dependent metasurface. The computed field distribution $E_{M}$ can be written as:

(1) $$E_{M}\left(x,y\right)\;=F^{-1}\left\{H\left(f_{x},f_{y}\right)\cdot F\left[E_{in}\left(x,y\right)\right]\right\},$$

where F represents a 2D spatial Fourier transformation, $F^{-1}$ represents a 2D inverse spatial Fourier transformation, and $H(f_{x},f_{y})$ is the optical spectrum transfer function, which is contributed by our metasurface. $f_{x}$ and $f_{y}$ are, respectively, *u*- and *v*-direction spatial frequency coordinates in the Fourier plane. Here, two different $H(f_{x},f_{y})$ are designed for dual-modal single-pixel sensing. When bright-field imaging mode is chosen, the $H(f_{x},f_{y})$ is equal to a constant. When edge-enhanced imaging mode is chosen, we utilize spiral phase contrast imaging based on the vortex beam [28] to design the $H(f_{x},f_{y})$. In this regard, $H(f_{x},f_{y})$ can be written as:

(2) $$H_{x}\left(f_{x},f_{y}\right)=\exp\left[i(\phi +C_{1})\right]$$

(3) $$H_{y}\left(f_{x},f_{y}\right)=\exp\left(iC_{2}\right)$$

where $H_{x}\left(f_{x},f_{y}\right)$ and $H_{y}\left(f_{x},f_{y}\right)$ are optical spectrum transfer functions for edge-enhanced (XLP) and bright-field imaging (YLP), respectively, $f_{x}=u/\left(\lambda f\right),$$f_{y}=v/\left(\lambda f\right),$ϕ=arctan(v/u), and $C_{m}\left(m=1,2\right)$ is constant. Our metasurface required phase profiles are $\Psi_{x}=\phi +C_{1}$ and $\Psi_{y}=C_{2}$, which are, respectively, under the illumination of an XLP and YLP incident beam. Next, the $E_{M}\left(x,y\right)$ field distributions for dual modes are both modulated by the corresponding patterns [34] projected onto the SLM. The obtained inner product $D_{j}$ between patterns $P_{j}\left(x,y\right)$ and $E_{M}\left(x,y\right)$ is measured by the single-pixel detector, which can be written as:

(4) $$D_{j}=\sum_{x}\sum_{y}P_{j}\left(x,y\right)E_{M}\left(x,y\right),\;j=1,2,\ldots ,n.$$

Accordingly, we finish the acquisition of the modulated target scene in dual modes. Then, the Alternating Direction Method of Multipliers (ADMM) framework is utilized to reconstruct the target image *O*. We introduce an auxiliary parameter *Q* to build the objective function into:

(5) $$\left(O,Q\right)={\mathrm{argmin}∥AO-D∥}^{2}+TV\left(Q\right),\;s.t.\;O=Q,$$

where $A\in R^{m\times n}$ denotes the modulation matrix (m modulation patterns, and each pattern consists of n pixels), and $D\in R^{m\times 1}$ is the measurement vector. In addition, TV(O) is the total variation regularization term. The following distributed sub-problems can solve the minimization in Equation (5),

(6) $$\begin{array}{cc} O^{k+1} & ={\mathrm{argmin}∥AO-D∥}^{2}+\frac{\rho }{2}{∥O^{k}-\left(Q^{k}-\frac{1}{\rho }W^{k}\right)∥}^{2},\\ Q^{k+1} & =\mathrm{argmin}TV\left(Q\right)+\frac{\rho }{2}{∥Q^{k}-\left(O^{k+1}+\frac{1}{\rho }W^{k}\right)∥}^{2},\\ W^{k+1} & =W^{k}+\rho \left(O^{k+1}-Q^{k+1}\right), \end{array}$$

where the superscript *k* represents the iteration number, and ρ represents the hyper-parameter. The sub-problem has a closed-form solution:

(7) $$O^{k+1}=O^{k}+\alpha A^{-1}\left(D-A\left(Q^{k+1}+W^{k+1}\right)\right),$$

where α represents the hyper-parameter. When the above equation converges, we can reconstruct the target’s bright-field or edge-enhanced images as requested.

In addition, we want to emphasize that different patterns correspond to different modulations, the main ones being Fourier, Hadamard, and random modulations. We will describe these modulations in detail next. It should be noted that this work mainly uses Fourier modulation, but this does not affect the generalization of our method. We also prove the generalization of the method in Section 3.3.

### 2.2. Fourier Modulation

Fourier modulation projects sinusoidal patterns to the target scene and captures the one-dimensional signal with the bucket detector [34]. The Fourier basic pattern *P* can be expressed as:

(8) $$P\left(x,y\right)=0.5+0.5*sin(2\pi \left(f_{x}+f_{y}\right)+\phi ),$$

where ϕ represents the initial phase. We use three-step phase shifting to sample images. Each coefficient in the Fourier space $F(f_{x},f_{y})$ is derived using three sinusoidal patterns with the same spatial frequency and different initial phases:

(9) $$F\left(f_{x},f_{y}\right)=\left(2D_{0}-D_{2\pi /3}-D_{4\pi /3}\right)+\sqrt{3}i\cdot \left(D_{2\pi /3}-D_{4\pi /3}\right),$$

where $D_{0},D_{2\pi /3}$, and $D_{4\pi /3}$ are the measurements corresponding to the illumination patterns of P(x,y,0),P(x,y,2π/3), and P(x,y,4π/3), respectively. Because of the conjugate symmetric feature of the Fourier spectrum, we only need to measure the upper half of the Fourier coefficients.

Then, the objective function can be transformed into:

(10) $$\left(O,Q\right)={\mathrm{argmin}∥F\left(O\right)-F∥}^{2}+TV\left(Q\right),\;s.t.\;O=Q,$$

The problems can be solved like Equations (6) and (7).

### 2.3. Hadamard Modulation

Hadamard modulation is based on Hadamard transform. By applying an inverse Hadamard transform to a delta function $\delta_{H}\left(u,v\right)$, the Hadamard transform pattern $P_{H}\left(x,y\right)$ can be obtained [3,35,36]:

(11) $$P_{H}\left(x,y\right)=\frac{1}{2}\left[1+H^{-1}\left\{\delta_{H}\left(u,v\right)\right\}\right],$$

where H and $H^{-1}\left\{\right\}$ denote a Hadamard transform and an inverse Hadamard transform, respectively.

(12) $$\delta_{H}\left(u,v\right)=\left\{\begin{array}{cc} 1, & u=u_{0},v=v_{0}\\ 0, & \;\mathrm{otherwise}\; \end{array}.\right.$$

Each Hadamard coefficient H(u,v) is acquired by two measurements. They are one measurement corresponding to a Hadamard basis pattern $P_{H}\left(x,y\right)$ and one measurement corresponding to an inverse pattern $\left[1-P_{H}\left(x,y\right)\right]$. The coefficient H(u,v) is obtained by using the two corresponding measurements:

(13) $$H\left(u,v\right)=D_{+1}-D_{-1}$$

where $D_{+1}$ and $D_{-1}$ are measurements corresponding to the illuminations of $P_{H}\left(x,y\right)$ and $\left[1-P_{H}\left(x,y\right)\right]$.

Then, the objective function can be transformed into:

(14) $$\left(O,Q\right)={\mathrm{argmin}∥H\left(O\right)-H∥}^{2}+TV\left(Q\right),\;s.t.\;O=Q,$$

The problems can be solved like Equations (6) and (7).

### 2.4. Binary Random Modulation

The binary random pattern $P_{j}$ is generated by a binary pseudo-random number generator [37]. This modulation method can realize the projection consistent with the speed of the SLM, because the number of bits is only one. It greatly increases the modulation speed. The objective function can be solved directly by referring to Equations (5)–(7).

## 3. Simulations and Analysis

### 3.1. Design of Metasurface

**Figure 2:**
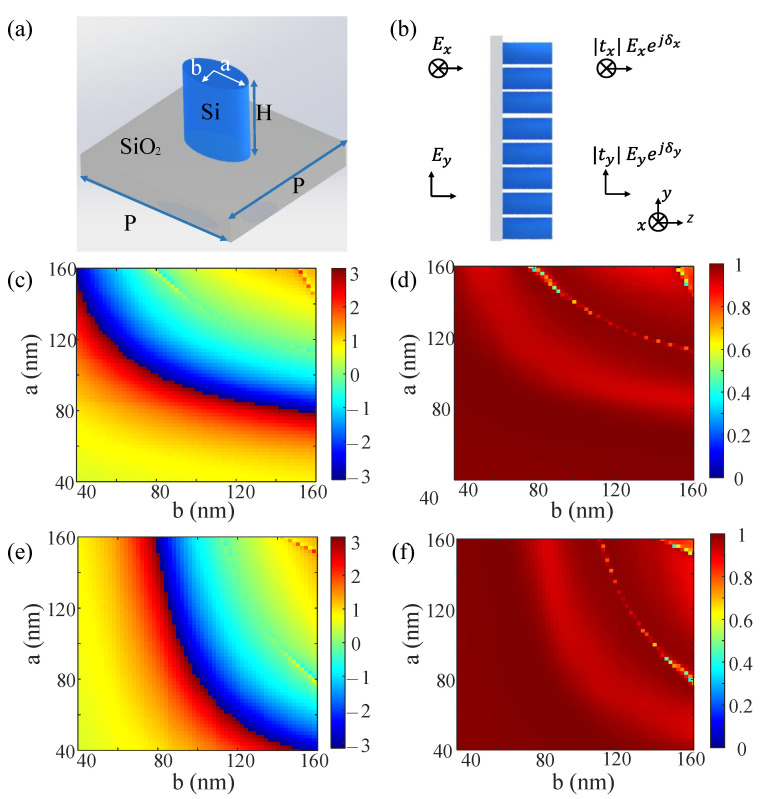
Design of the polarization-dependent metasurface. (**a**) is a side view of a typical unit cell with the period (*P*), height (*H*), and varying cross sizes (*a* and *b*). (**b**) is schematic for the numerical calculation of the transmission coefficients ($t_{x},t_{y}$) and phase shifts ($\delta_{x},\delta_{y}$). (**c**,**d**) are simulated propagation phase $\delta_{x}$ and transmission coefficient distribution $t_{x}$ for an XLP incident beam. (**e**,**f**) are simulated propagation phase $\delta_{y}$ and transmission coefficient distribution $t_{y}$ for an YLP incident beam.

To realize the proposed dual-modal metasurface-based device, we designed the dual-functional metasurface mentioned in Section 2. When the polarization of the incident beam is XLP, the designed metasurface should work as an edge filter in the Fourier plane. When the polarization of the incident beam is YLP, the designed metasurface should work as a bright-field filter in the Fourier plane. We used the polarization-dependent propagation phase of the nanobrick to achieve the two different phase profiles mentioned above, $\Psi_{x}$ and $\Psi_{y}$. The simulation results were calculated through the software-FDTD solutions, where periodic boundary layers are used in the *x* and *y* directions, and a perfectly matching layer is used in the *z* direction. The mesh accuracy is equal to 2. The distance of the monitor from the nanobricks is 3 μm. Dispersion is included in the material data. The plane-wave sources are utilized. Figure 2a displays a side view of a specially designed unit cell consisting of a silicon nanobrick sitting on a fused silica substrate. These nanobricks are periodically arranged with a fixed square lattice constant P=360nm and a height H=500nm. The propagation phase can basically cover 0∼2π by changing the major semi-axis *a* and minor semi-axis *b* of the nanobrick. The schematic for computing the transmission coefficients ($t_{x},t_{y}$) and phase shifts ($\delta_{x},\delta_{y}$) is shown in Figure 2b.

The simulated phase distribution $\delta_{x}$ for an XLP incident beam as a function of *a* and *b* is shown in Figure 2c, and the corresponding transmission coefficient distribution $t_{x}$ is exhibited in Figure 2d. Similarly, the simulated phase distribution $\delta_{y}$ for a YLP incident beam is shown in Figure 2e, and the corresponding transmission coefficient distribution $t_{y}$ is exhibited in Figure 2f. Accordingly, we chose an appropriate size (a,b) of the nanobrick in theory to obtain phase combination $\left(\delta_{x},\delta_{y}\right)$, which is equal to $\left(\Psi_{x},\Psi_{y}\right)$. We chose 16 discrete phases in the range of 0∼2π, and picked 16 corresponding nanobricks based on the minimum phase difference with the phase in our library and the maximum transmittance coefficient. The 16 selected nanobricks for constructing the metasurface mentioned in Section 2 are shown in Table 1. The designed metasurface consists of 109 nanobricks along both *x* and *y* directions. The working wavelength of the incident laser is 0.9 μm.

**Figure 3:**
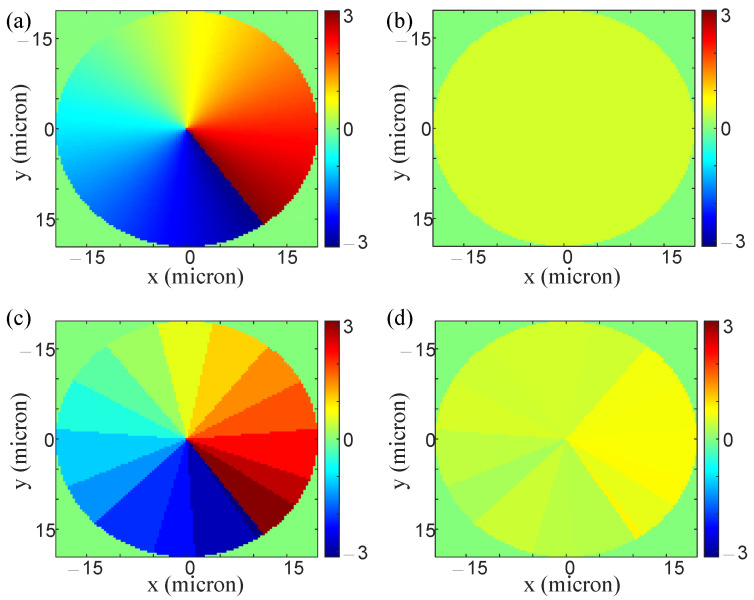
(**a**,**b**) are ideal phase profile distributions of the designed metasurface when illuminated by an XLP or YLP incident beam. (**c**,**d**) are real phase profile distributions of the designed metasurface when illuminated by an XLP or YLP incident beam.

**Figure 4:**
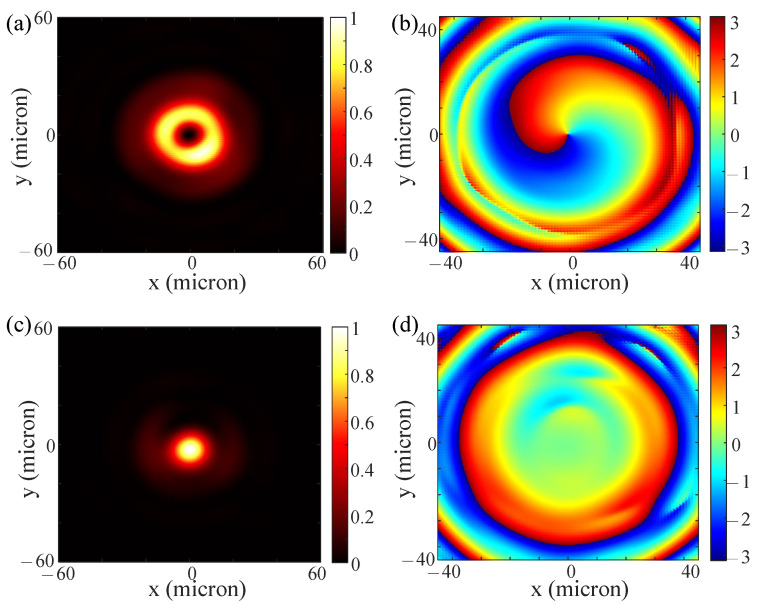
(**a**,**b**) are, respectively, simulated intensity and phase distribution of our designed metasurface under the illumination of an XLP incident beam. (**c**,**d**) are, respectively, simulated intensity and phase distribution of our designed metasurface under the illumination of a YLP incident beam.

To illustrate that the selected nanobricks are appropriate, we calculated the ideal and real phase profile distributions of the designed metasurface. Figure 3a shows the ideal phase profile distribution $\Psi_{x}$ of the designed metasurface under the illumination of the XLP. Figure 3b shows the ideal phase profile distribution $\Psi_{y}$ of the designed metasurface when illuminated by the YLP. Figure 3c,d display our designed metasurface’s real phase profile patterns under the illumination of the XLP or YLP incident beam. The phase distributions in Figure 3a,c are largely similar, albeit not entirely consistent, due to the selection of only 16 different nanobricks. However, these deviations do not significantly impact the function, as the spiral characteristic of the designed phase profile is well maintained. Similarly, the phase distributions in Figure 3b,d exhibit some inconsistencies due to the limitations of the nanobricks’ types, but these deviations are within acceptable tolerances. Other simulated results are shown in Figure 4. Respectively, Figure 4a,b show the far field intensity and phase distribution of the designed metasurface when illuminated by the XLP plane wave. Figure 4c,d, respectively, display the far field intensity and phase distribution of this metasurface when illuminated by the YLP plane wave. The doughnut-shaped intensity distribution is transformed into the Gaussian intensity distribution by altering the polarization of the incident beam. The phase pattern converts from a spiral-like distribution to an approximate constant distribution in the central area when switching the polarization of the incident beam. These figures show that our polarization-dependent metasurface is well designed.

### 3.2. Full-Process Simulations

**Figure 5:**
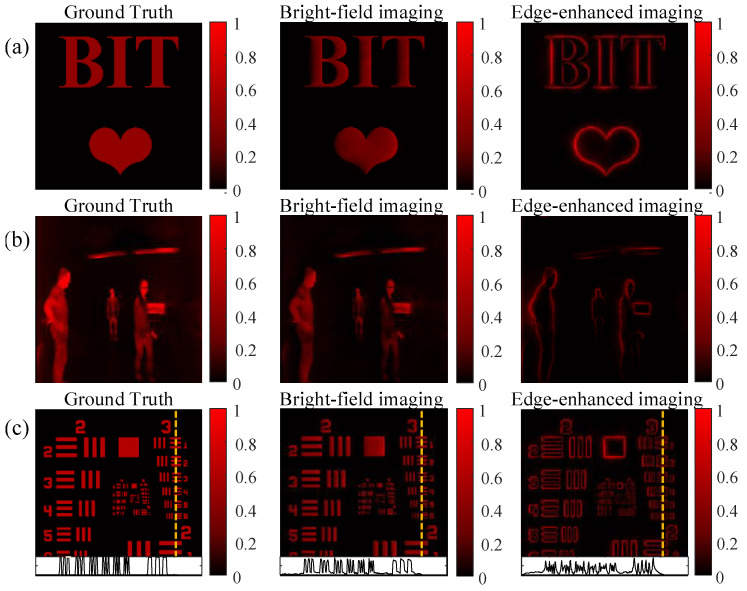
Metasurface-based computing results of bright-field filtering and edge-enhanced filtering. (**a**–**c**) are computing results using a “BIT” plus cardiogram image, an infrared image, and USAF.

To validate the implementability of our proposed device, we simulate the whole processing mentioned in Section 2. Based on MATLAB and FDTD-solutions, we first simulate the optical computing in Equation (1). This modulation is contributed by a 4f system embedded in our designed metasurface. Its computing result is shown in Figure 5. The input images are, respectively, a “BIT” plus cardiogram image, an infrared image [38], and USAF. Specifically, when the polarization of the incident beam is XLP, the designed metasurface should work as an edge filter in the Fourier plane. When the polarization of the incident beam is YLP, the designed metasurface should work as a bright-field filter in the Fourier plane. These bright-field filtering outputs are generally fainter than the ground truth but acceptable when illuminated by the YLP incident beam. All edge information after filtering is obvious when illuminated by the XLP incident beam. Moreover, Figure 5c shows details of the orange line on USAF, which maintains sharp textures and enhances the edge information. It could be concluded that optical computing based on the designed metasurface can efficiently achieve a dynamic switch between bright-field filtering and edge-enhanced filtering at 0.9 μm, by changing the polarization of the incident beam.

**Figure 6:**
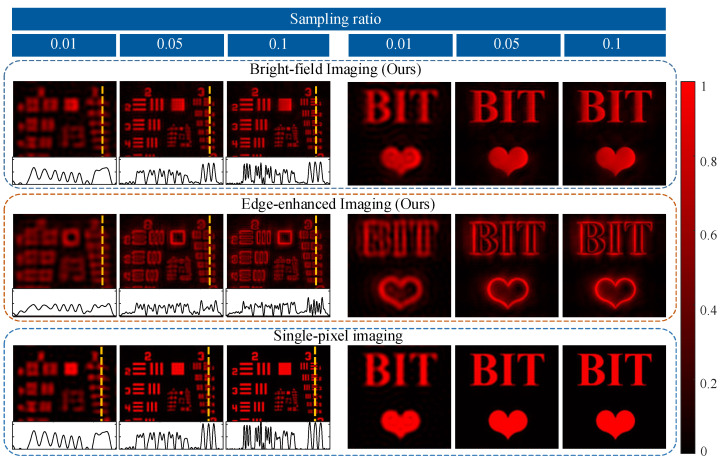
The proposed dual-modal single-pixel sensing of our device and conventional single-pixel imaging [34].

Next, we couple these intermediate results with the corresponding Fourier basis patterns and reconstruct the relevant images [256, 256] in dual modes. We obtain the final results at different sampling ratios and compare them with those of conventional single-pixel imaging [34], as shown in Figure 6. Imaging results are generally fainter than conventional single-pixel imaging, but overall details are still well maintained. As the sampling ratio rises, the structures and details are recovered more clearly. The details of the orange line on USAF show that the device could maintain sharp textures and edge information at a low sampling ratio. It could be concluded that our proposed dual-modal metasurface-based device can indeed achieve high-fidelity dual-modal sensing.

### 3.3. Generalization Analysis

**Figure 7:**
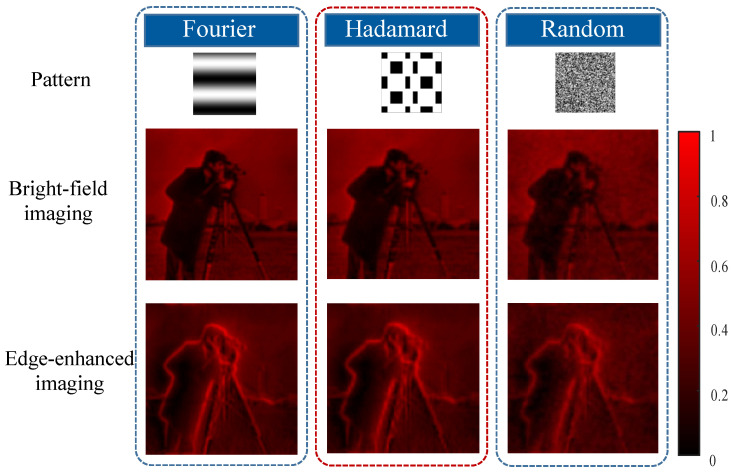
The proposed dual-modal single-pixel sensing results of our device under different modulations.

In addition, we also investigate the generalization of our dual-modal device in modulation. As shown in Figure 7, we applied Hadamard patterns [3,35,36], Fourier patterns [34], and random binary patterns [37] to couple the target information in two different modes. We obtained 6554 measurements of the cameraman image [128, 128] in the above modulations. From these figures, we can find that all of these bright-field and edge-enhanced images can be well reconstructed. Although the random method is slightly worse than the other modulations, these results still illustrate that various patterns are applied to our device.

### 3.4. Robustness Analysis

**Figure 8:**
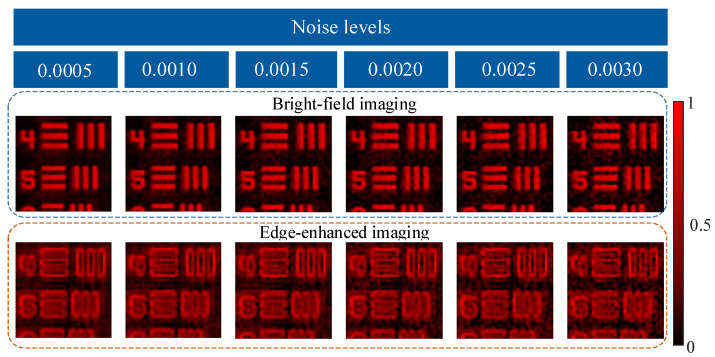
The proposed dual-modal single-pixel sensing results of our device under different noise levels.

In practical scenarios, various factors such as dark current and thermal noise usually affect the imaging quality. To further perform an actual scene, we simulated the whole acquisition and reconstruction processing under different noise levels. Gaussian white noise was added to the one-dimensional measurements. The sampling ratio was 10%. As shown in Figure 8, the reconstructed images basically maintain most details under different noise levels. Edge-enhanced images are more sensitive to noise than bright-field images because the frequencies of edge-enhanced images are concentrated on high-frequency bands. In brief, despite the relatively high noise level, our device can still obtain acceptable edge-enhanced or bright-field images. Therefore, we think our proposed device has certain noise robustness.

**Figure 9:**
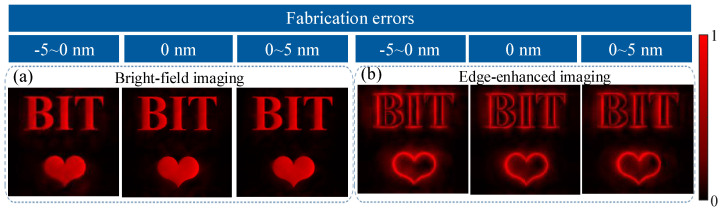
The proposed dual-modal single-pixel sensing of our device under different fabrication errors. (**a**) shows results of bright-field imaging under different fabrication errors, (**b**) shows results of edge-enhanced imaging under different fabrication errors.

In addition, random errors may arise during the processing of the metasurface due to unstable factors such as the environment. Robustness analysis is important as these random errors may result in changes in or even the deterioration of the experimental results. Simulating the process can help analyze the robustness and set reasonable accuracy requirements for the actual processing of the metasurface. During the fabrication, the central position of the nanobrick is relatively accurate, while its shape is not easily accurate and has a relatively great influence on phase modulation. Therefore, we kept the height of the nanobricks constant and added random errors mainly to the major and minor axes of each nanobrick. Three simulations were conducted with random errors ranging from −5 to 0 nm, 0, and 0 to 5 nm for each simulation to observe the results under different error ranges. The sampling ratio was 10%. The bright-field image obtained when the polarization of the incident beam is YLP is shown in Figure 9a, and the edge-enhanced image obtained when the incident beam is XLP is shown in Figure 9b. Our findings show that the simulation results under different ranges of random errors are various, but it is not very obvious. The overall image quality is still acceptable, indicating that our metasurface design can tolerate certain processing errors.

## 4. Biomedical Applications

In biomedical diagnosis, segmenting cell substructures allows for the analysis of clinical parameters related to volume and shape [39]. Bright-field imaging is high in redundancy but beneficial for complex segmentation tasks. However, medical images usually have low contrast and complex microstructure distributions, so edge image acquisition is one of the critical technologies in medical image processing. For segmentation, edge-enhanced images containing morphologies [40,41] are used to confirm the target’s boundaries. Many researchers have considered the meaning of edge enhancement in medical segmentation, which has effectively improved the segmentation accuracy through methods such as Edge Attention Network (ET-Net) [40], and KiU-Net [41]. These all demonstrate the important role of edge information in medical segmentation. Our device containing optical computing can directly output these bright-field and edge-enhanced images. It can work as the front end of neural networks for extracting features and directly provides edge information for network training, thereby reducing training parameters and computation.

**Figure 10:**
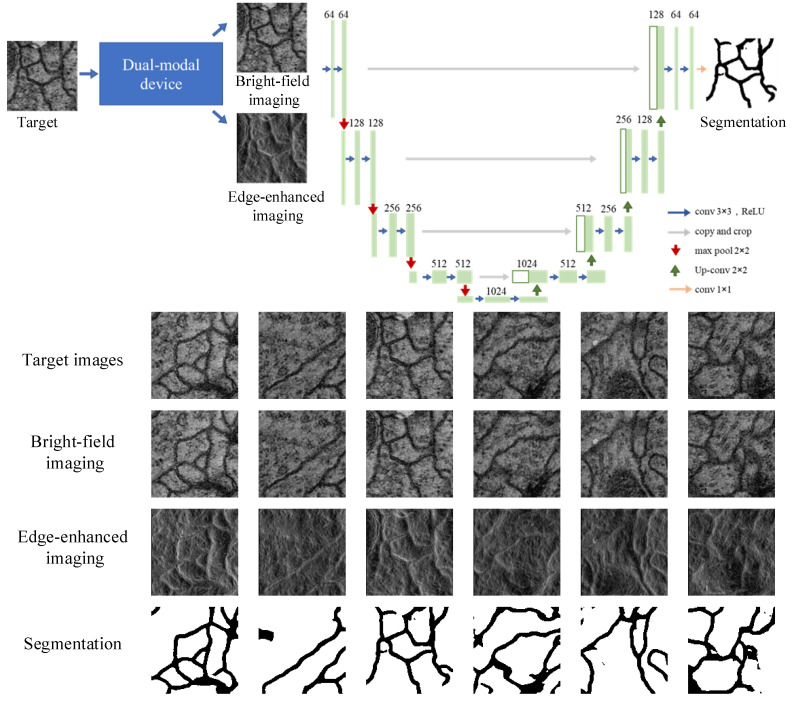
Single-pixel segmentation flowchart and corresponding results.

To validate that our device can be smoothly combined with the neural network, we used biomedical images modulated by our device to train the classical Unet [42] for cell segmentation. The initial dataset is from serial section transmission electron microscopy of the Drosophila first instar larva ventral nerve cord (VNC) [43]. It contains 30 training images, whose sizes are [512, 512]. We expanded the dataset to 1200 images by randomly cropping the original training images into [128, 128] pixels. In this dataset, the corresponding ground truth segmentation results are manually sketched, where the cells and membranes are marked in white and black, respectively. The framework of Unet is shown in Figure 10, which has a contracting path to capture context and a symmetric expanding path that enables precise localization. We used the normalization initialization method with the bias initialized to 0 and used the Adam solver for gradient optimization. The weight decay was 1 $\times \;10^{-3}$ and was decreased by 0.1 at 50 epochs and 350 epochs. We utilized Cross-Entropy loss, BCE loss, and Dice loss to train Unet. We obtained segmentation results of cells and membranes when we input these corresponding bright-field and edge-enhanced images into the trained Unet. Figure 10 validates that the outputs of our device could be efficiently applied to extract target features. We believe that the combination of bright-field and edge-enhanced images provides a low-power consumption approach for medical segmentation under a rationally designed network.

## 5. Conclusions

In summary, we report a near-infrared dual-modal single-pixel sensing device based on a polarization-dependent metasurface, which realizes switchable edge-enhanced imaging and bright-field imaging. Results show that it can achieve high-fidelity dual-modal single-pixel sensing at 0.9 μm and has certain noise robustness. We explored the proposed device’s potential in biomedical image analysis. The advantages of the dual-modal device lie in three aspects. First, this device realizes dual-modal sensing in a simple optical system. By rotating a polarizer to change the polarization of the incident beam, the designed polarization-dependent metasurface can function as different filters for different sensing tasks. Second, this novel device could be applied to all illumination patterns, which differs from the existing edge single-pixel methods. It maintains generalization ability on different illumination patterns. The same illumination patterns can be utilized for dual modes, even different sensing tasks. These advantages will not limit further optimization for high-accuracy imaging. Third, the optical analog computing based on our designed metasurface can process target scenes with high speed and low-power consumption. Moreover, these kinds of devices containing optical computing have widely worked as the front end of neural networks for extracting required information from high-redundancy target scenes. Therefore, we can envision that our device, combined with the neural network, can pave a new path for further intriguing sensing tasks. 

## Figures and Tables

**Table 1:** Metasurface parameters of the 16 selected nanobricks.

NUM	a(nm)	b(nm)	$\delta_{x}$	$t_{x}$	$\delta_{y}$	$t_{y}$
1	78	160	3.07	0.85	0.62	0.4
2	82	156	−2.81	0.83	0.32	0.74
3	86	152	−2.32	0.83	0.36	0.68
4	88	150	−2.11	0.88	0.44	0.53
5	92	160	−1.46	0.92	0.23	0.95
6	96	160	−1.10	1	0.38	0.94
7	104	40	1.48	0.96	0.69	0.98
8	104	158	−0.60	0.96	0.53	0.91
9	112	152	−0.24	0.99	0.49	0.91
10	116	40	1.86	0.95	0.72	0.98
11	126	144	0.18	0.98	0.50	0.92
12	130	40	2.33	0.97	0.76	0.98
13	140	40	2.73	0.9	0.79	0.98
14	144	136	0.62	0.96	0.50	0.92
15	152	40	−3.14	0.78	0.82	0.98
16	160	130	1.03	0.85	0.47	0.92

## Data Availability

Data underlying the results presented in this paper are not publicly available at this time but may be obtained from the authors upon reasonable request.
